# Comparative in silico genome analysis of *Clostridium perfringens* unravels stable phylogroups with different genome characteristics and pathogenic potential

**DOI:** 10.1038/s41598-021-86148-8

**Published:** 2021-03-24

**Authors:** Mostafa Y. Abdel-Glil, Prasad Thomas, Jörg Linde, Anne Busch, Lothar H. Wieler, Heinrich Neubauer, Christian Seyboldt

**Affiliations:** 1 Institute of Bacterial Infections and Zoonoses, Friedrich-Loeffler-Institut, Naumburger Str. 96A, 07743 Jena, Germany; 2 Department of Pathology, Faculty of Veterinary Medicine, Zagazig University, Zagazig, Sharkia Province Egypt; 3 Robert Koch-Institut, Nordufer 20, 13353 Berlin, Germany; 4 Institute of Microbiology and Epizootics, Department of Veterinary Medicine, Freie Universität, Robert-von-Ostertag-Str. 7-13, Building 35, 14163 Berlin, Germany; 5 Present Address: Division of Bacteriology and Mycology, ICAR-Indian Veterinary Research Institute, Izatnagar, Bareilly, 243122 India; 6 Present Address: Department of Anaesthesiology and Intensive Care Medicine, University Hospital Jena, Am Klinikum 1, 07747 Jena, Germany

**Keywords:** Microbiology, Bacterial genetics, Phylogenetics, Bacterial infection

## Abstract

*Clostridium perfringens* causes a plethora of devastating infections, with toxin production being the underlying mechanism of pathogenicity in various hosts. Genomic analyses of 206 public-available *C.* *perfringens* strains´ sequence data identified a substantial degree of genomic variability in respect to episome content, chromosome size and mobile elements. However, the position and order of the local collinear blocks on the chromosome showed a considerable degree of preservation. The strains were divided into five stable phylogroups (I–V). Phylogroup I contained human food poisoning strains with chromosomal enterotoxin (*cpe*) and a Darmbrand strain characterized by a high frequency of mobile elements, a relatively small genome size and a marked loss of chromosomal genes, including loss of genes encoding virulence traits. These features might correspond to the adaptation of these strains to a particular habitat, causing human foodborne illnesses. This contrasts strains that belong to phylogroup II where the genome size points to the acquisition of genetic material. Most strains of phylogroup II have been isolated from enteric lesions in horses and dogs. Phylogroups III, IV and V are heterogeneous groups containing a variety of different strains, with phylogroup III being the most abundant (65.5%). In conclusion, *C.* *perfringens* displays five stable phylogroups reflecting different disease involvements, prompting further studies on the evolution of this highly important pathogen.

## Introduction

*Clostridium (C.) perfringens*, a Gram-positive anaerobic and spore-forming bacterium, is found ubiquitously in the environment and the gut of humans and animals ^1^. This bacterium infects humans and livestock and produces a large number of extracellular toxins. Syndromes caused are gas gangrene, enteritis and enterotoxaemia ^1,2^. Six key toxins (α, β, ɛ, ɩ, enterotoxin and *netB*) are used to categorize *C.* *perfringens* into seven toxin types (A to G) ^2^. Yet, more than 20 toxins and enzymes contribute to the virulence of *C. perfringens* ^1^. Type A *C.* *perfringens* causes enteric infections in various hosts and is also involved in cases of histotoxic infections where α-toxin is thought to be the key virulence factor and that perfringolysin (PFO) acts synergistically with α-toxin to cause progressive tissue damage ^1,3^. *C. perfringens* type B secretes β- and ɛ- typing toxins, and causes enteritis and enterotoxaemia in various animal species ^3^. *C. perfringens* type C produces β- toxin but may also express other plasmid-encoded toxins such as enterotoxin, beta2 and TpeL but not ɛ-toxin ^2^. Type C diseases in animals include hemorrhagic necrotizing enteritis in lambs, piglets, calves and foals. The newborn animals are typically the most susceptible especially piglets ^3^. Type D secretes the ɛ toxin, a highly potent clostridial toxin. Diseases caused by type D strains are among the most common clostridial diseases in sheep and goats and are sometimes referred to as “pulpy kidney disease”, characterized by sudden death or neurological and respiratory signs ^3^. *C. perfringens* type E produces ɩ-toxin, a clostridial binary toxin which is encoded by two plasmid genes. Type E strains are commonly isolated from cases of hemorrhagic enteritis and sudden death in neonatal calves and are infrequently found in lambs with enterotoxaemia ^4^. *C.* *perfringens* type F strains produce enterotoxin (CPE), a member of the aerolysin pore-forming toxin family, which is mostly associated with cases of human food poisoning, antibiotic-associated diarrhoea and sporadic non-foodborne illness ^5^. The gene encoding CPE (*cpe*) can be located on the chromosome or a plasmid. While plasmid-encoded *cpe* was also reported in type C, D and E strains ^4^, the chromosomally-encoded *cpe* was only detected in one group belonging to type F strains ^4^. *C. perfringens* type G includes strains that produce NetB toxin that is supposed to mainly cause poultry necrotic enteritis disease ^2^.

*C. perfringens* food-poisoning ranks among the most common bacterial foodborne diseases in the United States ^4,6^. Specific association of chromosomal *cpe* strains with food poisoning were reported in more than 70% of the cases ^4,6^ as these strains can survive (improper) cooking and replicate very fast in the food matrix ^4,5^. After ingestion, they can also survive the acidity of the stomach and passage to the intestine where they undergo sporulation and CPE production ^4^ thereby inducing lesions in the intestine, diarrhoea and abdominal cramps ^4,6^. The disease is relatively mild with an incubation period of 8–22 h ^4^. Unusual fatal outbreaks due to type F strains were also recorded ^7^. The characteristics of *C.* *perfringens* type F strains especially aspects of CPE toxicity and genetics were reviewed recently ^4^. Moreover, humans can be affected by *C.* *perfringens* type C also known as “Darmbrand” or “pigbel”. Infections caused by type C are rare and most reports describe the disease in individuals with reduced pancreatic functionality such as people with chronic illness ^8^, diabetic patients ^9–12^ and vegetarians who suddenly change to a diet rich in proteins ^13^. However, historical epidemics were described in Northern Germany in 1949 ^14^ and the highlands of Papua New Guinea in the 1960s ^15^. Type C enteritis necroticans is life-threatening in humans and characterized by hemorrhagic, inflammatory or ischemic necrosis of the jejunum associated with abdominal pain and severe bloody diarrhoea ^16^. To the authors’ knowledge, the complete genome sequence of a Darmbrand strain has not yet been analysed. In addition, relatively limited information is currently available about the genomes of type F food poisoning strains with chromosomal-encoded *cpe*. However, multi-locus sequence typing (MLST) indicated that chromosomal *cpe* strains and Darmbrand strains are phylogenetically-related ^17,18^. Additionally, two very recent genomic studies described the clonal relatedness of the chromosomal *cpe* strains ^19,20^. The current knowledge of *C.* *perfringens* genomics, virulence factors, toxins and antimicrobial potentials is described in a recent review ^1^.

A whole-genome sequence (WGS) analysis of 56 *C.* *perfringens* strains revealed a highly divergent open pangenome and indications of significant horizontal gene transfer ^21^. In addition, genome analysis of necrotic enteritis strains from poultry identified pathogenic clades based on the content of accessory genes of the strains, demonstrating a major role of accessory genes in the pathogenicity of *C. perfringens* ^22^.

A recent collaborative project between Public Health England, Wellcome Trust Sanger Institute and Pacific Biosciences was launched to sequence 3,000 bacterial genomes from the strain collection of the National Collection of Type Cultures ^23^. These sequence data are publicly available and include data of 23 NCTC *C.* *perfringens* strains including 13 food poisoning strains with chromosomal *cpe* (type F) and one type C Darmbrand strain. Our study aimed to assemble the sequence data of 23 NCTC strains that were sequenced within the framework of the NCTC 3000 project ^23^. Additionally, we combined these data along with 183 NCBI publicly-available genomes with the aim to investigate the chromosome variability and structure of the closed genomes (n = 34), as well as to investigate the phylogenetic structure and potential virulence capabilities between all strains (n = 206).

## Results and discussion

### Genomic overview and chromosomal (re)arrangement in *C. perfringens*

In order to investigate the *C.* *perfringens* genomic diversity, we acquired and assembled PacBio data of 23 NCTC strains sequenced by the NCTC 3000 project ^23^ (Table 1, Supplementary Table S1). The de novo assembly yielded 20 circularized chromosomes with a mean final coverage of 186.8 ± 42.3X (Data set 1 at https://doi.org/10.6084/m9.figshare.12264497) and a panel of 45 extrachromosomal elements, 32 of them were circularized (see methods, Table 1). The 23 assemblies based on PacBio sequences were combined with 183 assemblies downloaded from NCBI, 32 of them were *netF*-positive strains derived from cases of foal necrotizing enteritis (n = 16) and canine hemorrhagic diarrhoea (n = 16) ^24,25^. These 206 strains originate from different ecosystems (humans, animals, foods and environment) of various continents (America, Europe, Asia and Australia) and span a time period from the 1920s to 2010s (Supplementary Table S1).

**Table 1:** Results of de novo genome assembly for PacBio sequence data of 23 NCTC *Clostridium perfringens* strains.

						Extrachromosomal elements											
				Chromosome		A		B		C		D		E		F	
Strain	SRA Run	Toxin-type	Toxin genes	Status	Size (Mb)	Status	Size (Kb)	Status	Size (Kb)	Status	Size (Kb)	Status	Size (Kb)	Status	Size (Kb)	Status	Size (Kb)
NCTC8678	ERR1377187	A	*cpa*	●	2.9	◐	38	◐	4.7								
NCTC8797	ERR1377188	F	*cpa, cpe*	●?	2.9	◐	25.3	◐	19								
NCTC13170	ERR1377189	A	*cpa*	●	3.29	–	–										
NCTC8503	ERR1407347	D	*cpa, etx*	◐	3.4 (6 ctgs*)	●§ (*etx*)	54.6	●	19.3								
NCTC2544	ERR1456745	A	*cpa*	●	3.18	–	–										
NCTC8799	ERR1466824	F	*cpa, cpe*	●	2.9	◐	63.1	◐	10.2	◐	7.8						
NCTC10612	ERR1588634	F	*cpa, cpe*	●	3.0	●	13.6	◐	8.8								
NCTC3182	ERR1599940	C	*cpa, cpb*	◐	3.5 (1ctg*)	●	49.4	●§ (*cpb*)	59.1	●	37.2						
NCTC8238	ERR1656456	F	*cpa, cpe*	●	2.9	●	56.6										
NCTC8246	ERR1656458	A	*cpa, cpb2*	●	3.3	●	54.3										
NCTC2837	ERR1656459	A	*cpa*	●	3.3	–	–										
NCTC8081	ERR1656460	C	*cpa, cpb, cpe*	●	3.1	●§ (*cpe*)	116.6	●	73.5	●§ (*cpb*)	67.9	●	58.1	●	53.7	◐	20.7
NCTC8247	ERR1674568	F	*cpa, cpe*	●	2.9	◐	57.02										
NCTC8239	ERR1681948	F	*cpa, cpe*	●	2.9	●	56.6										
NCTC9851	ERR1681949	F	*cpa, cpe*	●	2.9	●	56.6	●	12.3								
NCTC10239	ERR1681950	F	*cpa, cpe*	●	2.9	●	57.9	●	12.4	●	12.2						
NCTC10240	ERR1681951	F	*cpa, cpe*	●	2.9	●	56.6	◐	9.7								
NCTC8679	ERR1787549	F	*cpa, cpe*	●	2.9	●	54.4	●	12.1								
NCTC10578	ERR1787550	A	*cpa*	●	3.3	–	–										
NCTC10613	ERR1787552	F	*cpa, cpe*	◐	2.94 (ctgs)	●	56.6	●	38.1	●	12.2	●	12.3	◐	8.9		
NCTC10614	ERR1800584	F	*cpa, cpe*	●	2.9	●	56.6	●	12.2								
NCTC8359	ERR1805687	F	*cpa, cpe*	●?	2.9	●	56.6	●	13.4	●	12.2						
NCTC11144	ERR1954484	F	*cpa, cpe*	●	3.2	●§ (*cpe*)	78	●	58.6	●	50.9						

*ctg; contig: a contiguous fragment of DNA.

● Circularized finished genetic element.

●? The genome is circularized but there is uncertainty about its finished status as deep valleys (assembly area with little support of PacBio reads) were noticed in the coverage plot of the genome of strain NCTC8797 that may indicate a misassembly issue. Also, the genome of strain NCTC8359 showed a large spike in the coverage plot which may indicate collapsed repeats.

◐ Draft genetic element.

●§; indicates possible conjugative plasmids carrying known significant toxin genes (epsilon, beta, entero-toxin genes) and the species conjugation locus.

**Figure 1:**
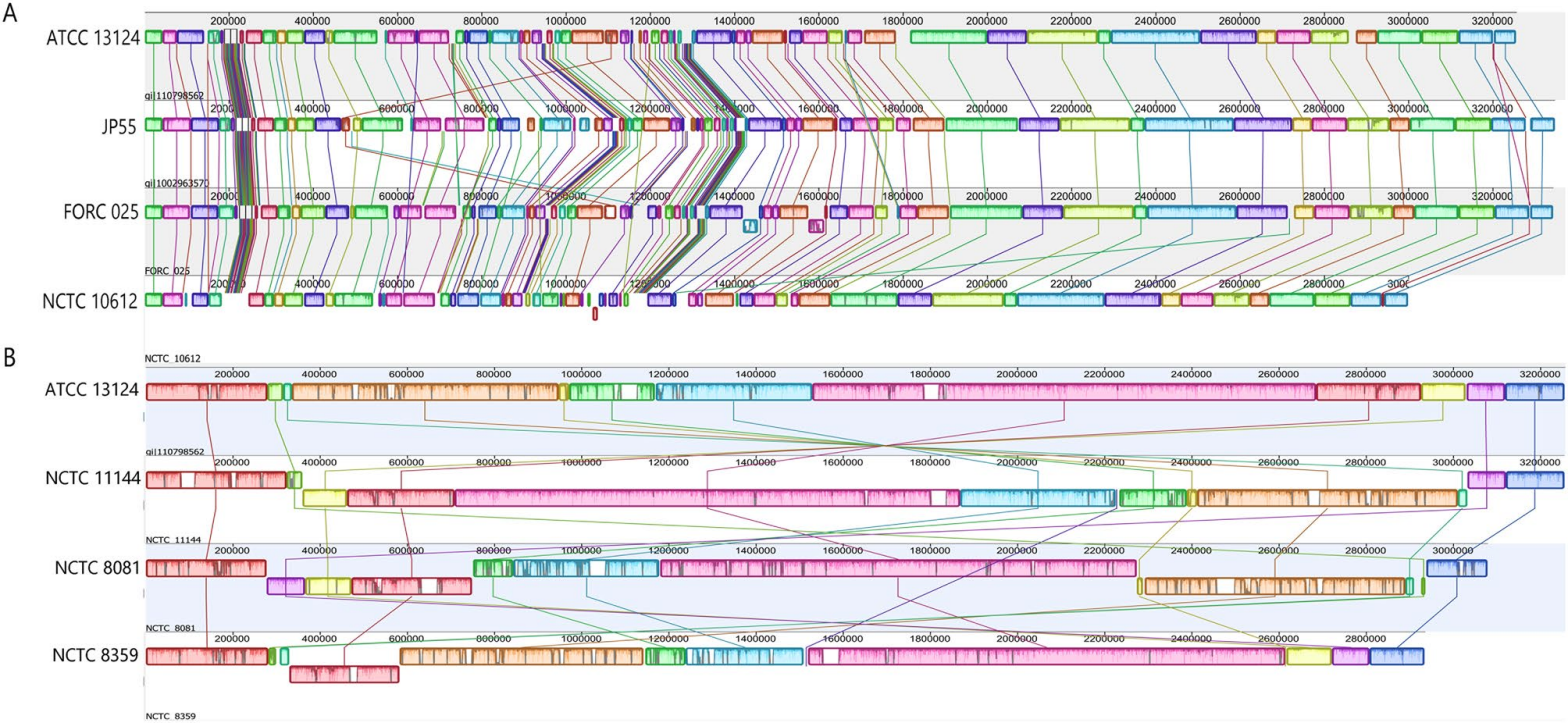
Whole-genome alignment of *Clostridium perfringens* strains. (**A**) A representative detail of the multiple genome alignment depicting genomic conservation within the strains in respect to the physical location of Locally Collinear Blocks (LCBs) and relative order of the genes. Please see Figure S1 for a detailed alignment of the 34 genomes. (**B**) Alignment of the *C. perfringens* genomes showing inversions and shifts (NCTC 11144, NCTC 8081 and NCTC 8359) relative to the reference strain (ATCC 13124). In strain NCTC 11144, a large inversion bordered by rRNA operons was observed while the inversions and shifts in the strains NCTC 8081 and NCTC 8359 were bordered by the IS element IS*Cpe7*. Position 1 in all strains corresponds to the origin of replication as determined in strain 13. The alignment was generated using progressiveMauve ^73^ and edited in Adobe Photoshop CS5 (www.adobe.com/photoshop).

Among the 206 analysed genomes, 34 were in a closed state of assembly (20 assembled in this study and 14 downloaded from NCBI; Supplementary Table S2; Data set 2 at https://doi.org/10.6084/m9.figshare.12264497). Considering these genomes, *C.* *perfringens* is composed of a circular chromosome of variable size (2.9–3.5 Mb) and up to six extrachromosomal elements. The food poisoning chromosomal *cpe* strains with circularized genomes (14 out of 34) have a consistently smaller chromosome size (≤ 3 Mb) compared to the other strains (> 3 Mb) as recently described ^20^. The *C.* *perfringens* chromosome contains on average 2,800 ± 187.8 (range 2,563–3,297) protein-coding sequences (CDS) (Supplementary Table S2). The calculated coding density (the size of coding regions over the genome size) was ~ 83%. The *C.* *perfringens* genome has low GC content (~ 28%) and carries ten rRNA operons except the type strain ATCC 13124 with eight rRNA operons (Supplementary Table S2). Plasmids of *C.* *perfringens* vary in size from 2.4 to 404 Kb and some of them harbour the conjugation locus of the species (transfer of clostridial plasmids; *tcp*) facilitating plasmid spread (Supplementary Table S2) ^26^. Plasmids contribute an average of 127.5 ± 151 (range 19–704) CDS i.e. up to 18% of the coding capacity of *C.* *perfringens* (Supplementary Table S2). These data suggest a substantial degree of variability among the genome content of *C.* *perfringens* and corroborate prior findings ^21^. However, despite this variability, there was general genomic conservation among the investigated strains with respect to the physical location and relative order of genes in each chromosome (Fig. 1, Supplementary Fig. S1). Few inversions mostly confined to integrated phages or genes flanked by IS elements were detected (Fig. 1A, Supplementary Fig. S1). However, strain NCTC 11144 (a food poisoning strain), NCTC 8081 (Darmbrand type C strain) and NCTC 8359 exhibited much less conservation in their genome organization with reversals and shifts that were observed along the genome segments (Fig. 1B). In these strains, the distribution of the PacBio reads across the chromosome showed no discontinuities in the mapping pattern. This might exclude the possibility of misassemblies in these genomes. However, a coverage spike observed in the strain NCTC 8359 was likely due to a repeat collapse (Data set 1 at https://doi.org/10.6084/m9.figshare.12264497).

Strain NCTC 11144 showed inversion of a large genomic region around the terminus of replication (Fig. 1B). This large inversion was bordered by rRNA operons. Large chromosomal inversions were already reported in various bacterial species to occur symmetrically around the replication origin and terminus ^27–29^ as it was previously suggested that most recombination events occur in relation to the replication fork ^30,31^. However, strains NCTC 8081 and NCTC 8359 do not follow the pattern of rearrangement of strain NCTC 11144. Two blocks of genes in strain NCTC 8081 were translocated and inverted whereas only one block in strain NCTC 8359 was translocated (Fig. 1B). The inversions and shifts in these two strains were bordered by identical copies of the IS element IS*Cpe7*. Chromosomal rearrangements in association with IS elements as found here have been also previously reported for example in *Bordetella* species. ^32^. These results—when taken together—imply a considerable conserved genomic synteny (physical location and relative order of homologous blocks) of ~ 90% (31 out of 34) of *C.* *perfringens* genomes*.* For unknown reasons, the frequency of large inversions and shifts seems to be very low in *C.* *perfringens* as well as in some other *Clostridium* species such as *C. botulinum* ^33^. Chromosome rearrangements can influence bacterial phenotype as found in *Escherichia coli* ^34^ and *Staphylococcus aureus* ^35^. However, it is unclear how these inversions influenced the phenotypic characteristics in *C.* *perfringens*.

### Impact of mobile genetic elements (MGE) on *C. perfringens* genome size and variability

**Figure 2:**
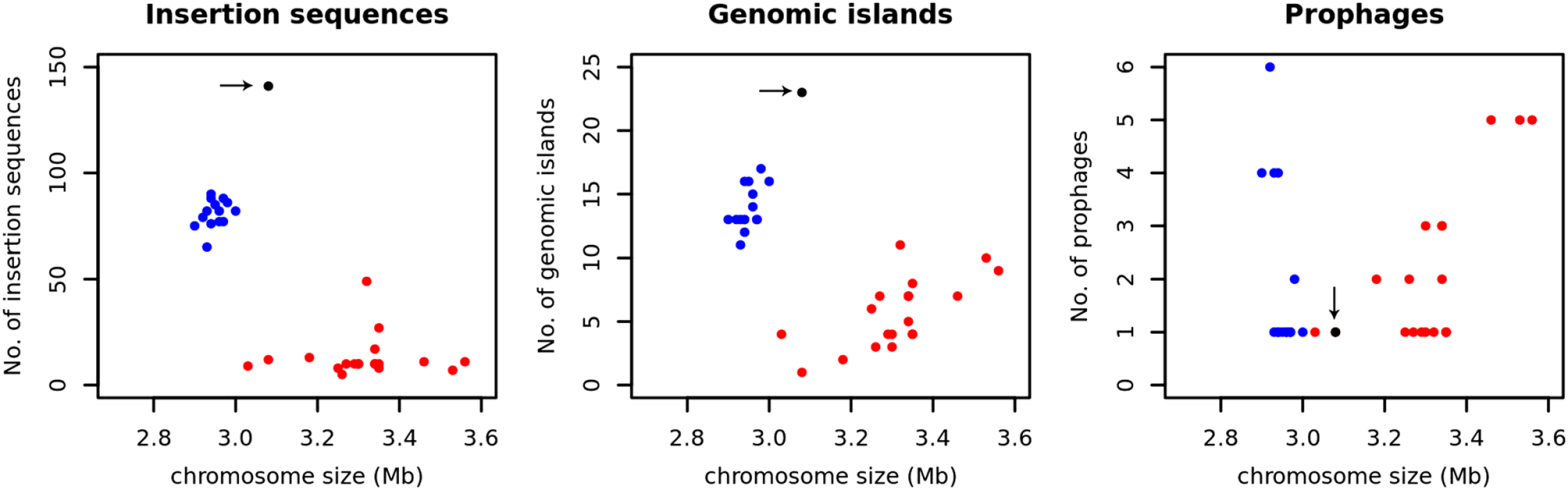
Mobile genetic elements in the closed chromosome of 34 *Clostridium perfringens* genomes. The predicted number of insertion sequences, genomic islands and prophages is plotted against the chromosome size. Human food poisoning strains carrying the enterotoxin gene on a chromosome (chromosomal *cpe* strains) are displayed in blue. Black colour (marked with an arrow) represents the Darmbrand (human enteritis necroticans) strain. Other strains are coloured red. The plots were generated in R ^87^.

**Figure 3:**
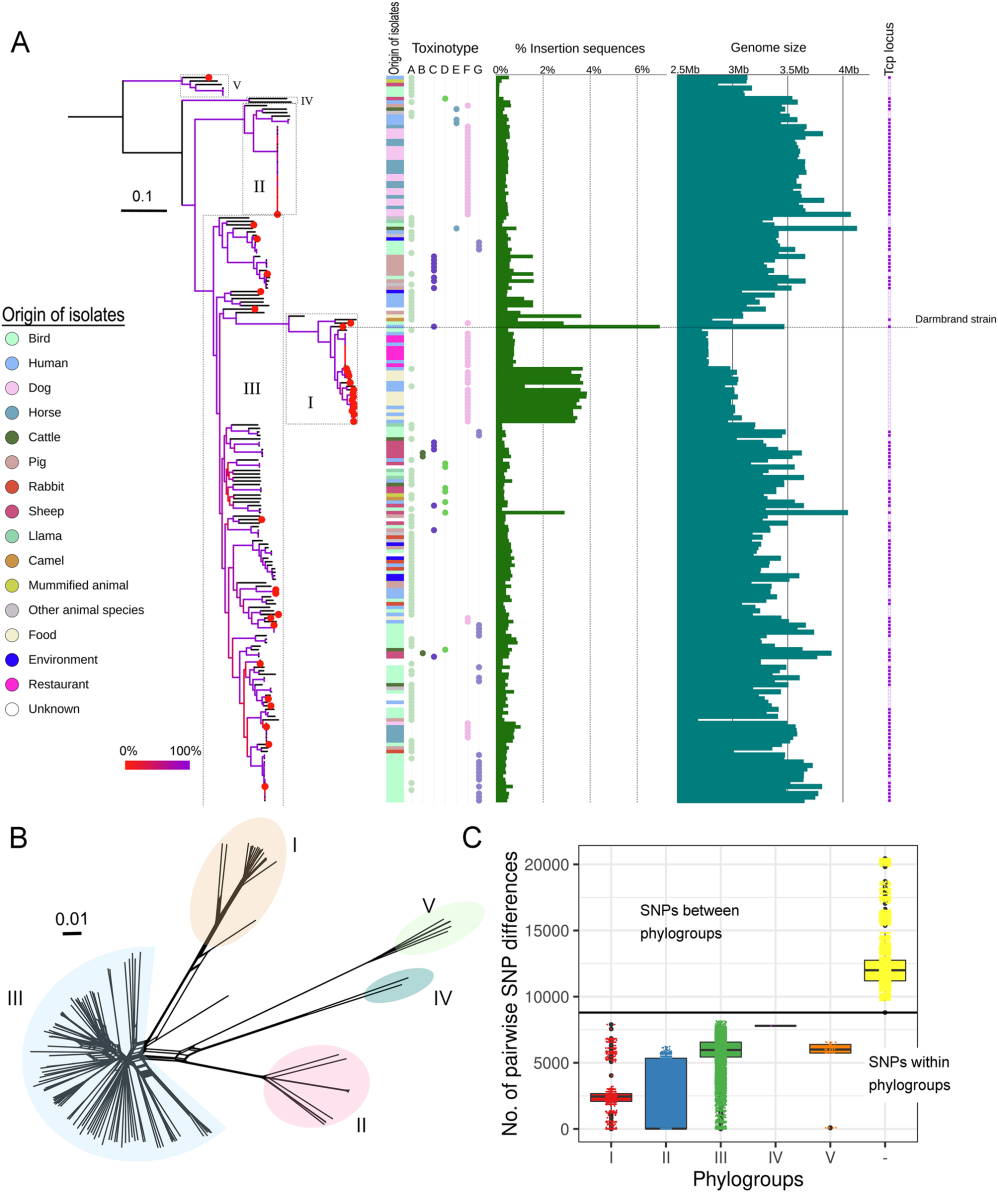
Phylogenetic relationships of 206 *Clostridium perfringens* genomes. (**A**) A maximum-likelihood (ML) phylogeny computed from the core genome SNPs using RAxML. Branch colouration denotes bootstrap values as described in the legend. Red colour coding at the tips of the tree indicate strains represented by closed genomes. The five major phylogroups are highlighted on the ML tree with boxes numbered I to V according to phylogroups described in the text. Strains’ origins and toxin types are plotted next to the ML tree. Bar plots represent the percentages of insertion sequences and the total size of the strains’ genomes, respectively. Genome size reported for closed genomes includes the chromosome and extrachromosomal elements. The presence (violet colour) or absence (white colour) of conjugation locus (*tcp*) in each strain is shown. The phylogenetic position of the historic Darmbrand strain is highlighted with a horizontal dotted line. The phylogenetic tree was visualized using iTOL ^81^ (**B**) SplitsTree phylogenetic network based on 63,036 unique SNP sites in the core genome of 206 *C.* *perfringens* strains with phylogroups highlighted. (**C**) The numbers of pairwise SNP distances between strains within each of the five phylogroups and between phylogroups are depicted. The plot was generated in R ^87^ and edited in Adobe Photoshop CS5 (www.adobe.com/photoshop).

MGE affect bacterial genome structure and function such as gene inactivation or activation, altering gene order and deletions of large DNA segments that may result in a reduction of genome size ^36–39^. We searched the closed genomes for the presence of MGE to detect integrated phages, insertion sequences and genomic islands (GI) as well as CRISPR elements, which confer protection against bacteriophage invaders in bacteria ^40^ (Supplementary Table S2). Prophages were detected at a variable range with no direct correlation between their frequency and the absence or presence of CRISPR elements (Supplementary Table S2). CRISPR elements were found in 18 strains but were absent in the chromosome of 16 strains (Supplementary Table S2). A CRISPR-*Cas* system of either class I (similar to class I-B described for *Clostridium klyveri*) or class II (similar to class II-C reported for *Neisseria lactamica*) was found in 17 strains (Supplementary Table S2) based on a recent CRISPR-*Cas* classification ^40^. Strain NCTC 10578 was predicted to harbour CRISPR-*Cas* systems of type I and type II as well as an additional CRISPR repeat flanked by a transposase gene (IS605 family) (Supplementary Table S2). IS elements and GIs were identified with highly variable occurrence between strains. We observed a high accumulation of ISs and GIs in the Darmbrand strain and in chromosomal *cpe* strains which are also characterized by a smaller genome size (< 3Mb) (Figs. 2, 3). In these strains, ISs and GIs constitute on average of 3.7% and 6.7% of the genome size, respectively in contrast to other strains (average genome size of 0.49% and 1.87% for IS and GI, respectively; Supplementary Table S2). According to our phylogenetic analysis, these genomes are closely-related forming a single phylogenetic group (referred to as phylogroup I) (Fig. 3). Several different IS families were observed in these genomes—IS200/IS605, IS6 and IS30 were primarily present (Supplementary Tables S2 and S3).

Previous genomic analysis of strain 13, SM101 and ATCC 13124 reported a skewed genomic variability towards one replichore ^41^. One of the genomes analysed (SM101) was enriched for IS elements which were unevenly distributed and biased to a more variable replichore ^41^. With the advantage of having 34 chromosomes in their closed state, we aimed to investigate the variability within these genomes to portray the distribution pattern of GIs and ISs across chromosomes. The alignment of the 34 completed genomes showed that the variable regions were present across the chromosome. However, their distribution was to some extent shifted toward one replichore with the exception of the three strains with different chromosomal rearrangements (Fig. 1, Supplementary Fig. S1). Locally collinear blocks (LCBs) within one replichore (left side in Supplementary Fig. S1) were shorter with several breakpoints and abundances of regions that lack detectable homology compared to the other replichore (right side in Supplementary Fig. S1). Plotting the distribution of IS elements and GIs across the chromosome revealed their asymmetrical distribution in the chromosomal *cpe* strains toward the less stable replichore (Supplementary Fig. S2). The Darmbrand strain was highly enriched in ISs and GIs (Fig. 2). However, because of DNA rearrangements a bias in IS and GI distribution was not observed (Supplementary Fig. S2). The concentration of IS elements and GIs towards one replichore is intriguing and could indicate that natural selection drives this distribution of IS elements in the chromosomal *cpe* strains. Genomic inversion patterns in *Bacillus* and *Clostridium* are reported to be dominated by symmetric inversions ^42^. However, an unanswered question in this respect is whether non-random genome organization is caused by random mutation processes in the context of replication or by selection ^42^.

### Distinct *C. perfringens* clustering based on core genome SNPs and accessory gene content

The genomes of 206 *C.* *perfringens* strains (34 closed and 172 non-closed genomes) were included to identify strain relationships based on the core genome and accessory gene content. For the core genome, we identified 63,036 SNPs in a core genome of 793,459 bp and used them to construct a maximum-likelihood (ML) tree (Fig. 3A, details Supplementary Table S4 and Data set 3 at https://doi.org/10.6084/m9.figshare.12264497) and a phylogenetic network (Fig. 3B). The core genome analysis grouped 206 strains into five major phylogroups (I—V) with 100% bootstrap support. These phylogroups could be additionally split into 114 clusters based on the tree patristic distance (Supplementary Fig. S3). The average number of SNP differences between genome pairs within the same phylogroup ranged from 1,909 to 7,792 SNPs, while the minimum SNP difference between genome pairs from different phylogroups was 8,796 SNPs (Fig. 3C).

**Figure 4:**
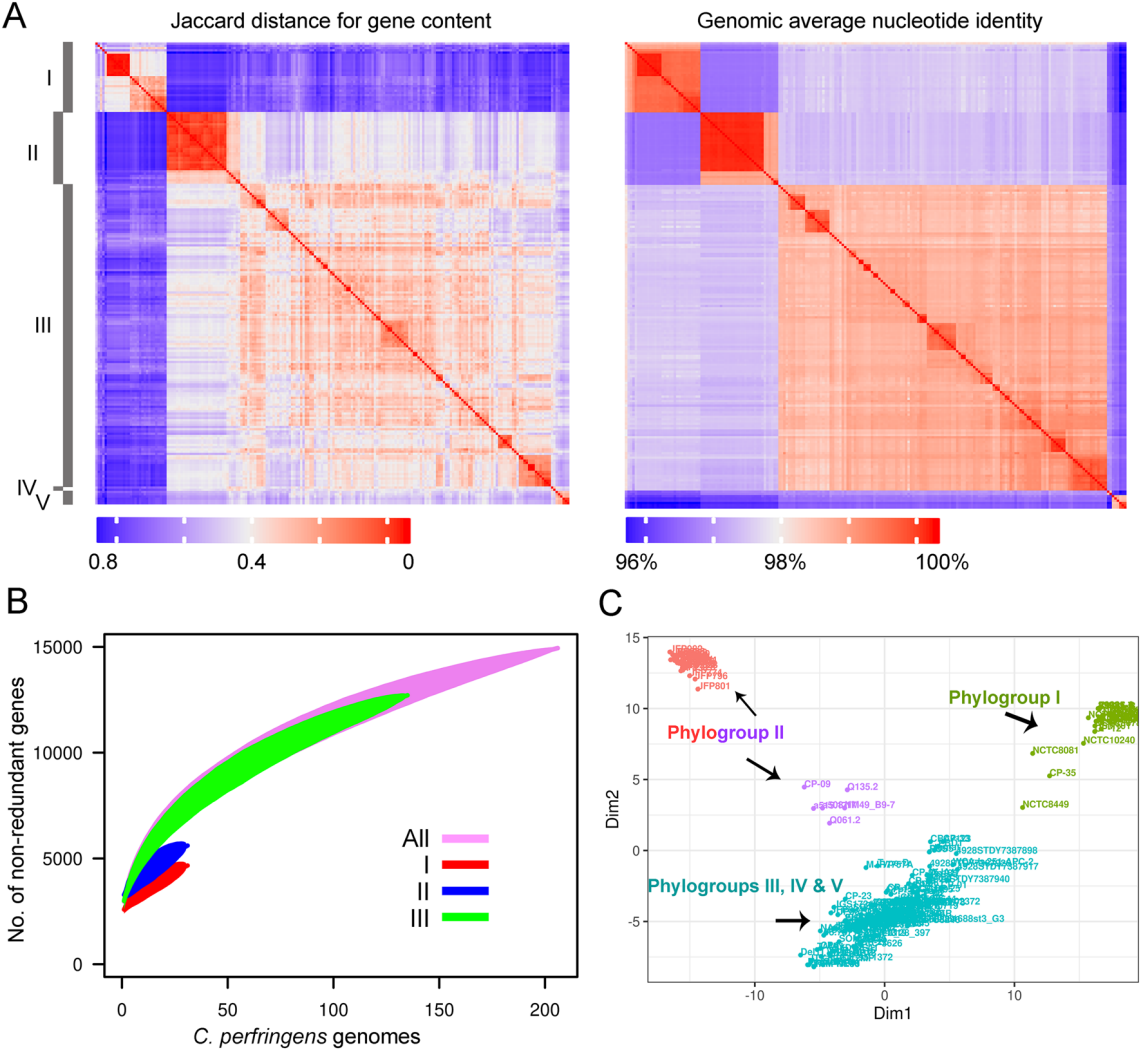
Accessory genome clustering and pangenome accumulation in 206 *Clostridium perfringens* strains. (**A**) Heat maps from similarity matrices calculated based on the distribution pattern of accessory genes (left) and the average nucleotide identity of the whole genome (right) in the 206 strains with phylogroups highlighted. Heat maps were generated using ComplexHeatmap ^91^ in R ^87^ and edited in Adobe Photoshop CS5 (www.adobe.com/photoshop). (**B**) Accumulation curves for the pangenome produced using vegan ^86^ in R ^87^ for the species (n = 206 strains, 14,942 genes) and the phylogroups I (n = 31 strains, 4,665 genes), II (n = 32 strains, 5,608 genes) and III (n = 135 strains, 12,713 genes). (C) Multidimensional scaling plot of the pangenome showing clustering of strains based on the pattern of presence and absence of accessory genes, with phylogroups highlighted and colour coded as calculated using the R function cmdscale () (goodness of fit = 0.3321971, X-axis eigenvalue = 0.2004366, and Y-axis eigenvalue = 0.1317606).

For the accessory genome, we identified 4,099 genes with a frequency of 2–95% in the strains. The pangenome of *C.* *perfringens* comprised 14,942 non-redundant protein-coding sequences and showed characteristics of an open pangenome (Fig. 4B, Data set 4 at https://doi.org/10.6084/m9.figshare.12264497). 8,808 genes (~ 59% of the pangenome) were present only in less than five strains. As for the species, the major phylogroups (I, II and III) had an open pangenome (Fig. 4B). The distribution pattern of the accessory genes led to the identification of three clusters that correlate with the major phylogroups from the core genome (Fig. 4C). Phylogroup I strains were distinctly separated based on the accessory gene content while strains of phylogroup II revealed two distinct patterns of accessory gene distribution. Strains of the phylogroups III to V clustered together based on the accessory gene content (Fig. 4C).

Phylogroup I comprised 31 strains mainly involved in cases of human foodborne diseases. These strains carried the *cpe* gene on a chromosome except the Darmbrand strain in which the gene was located on a 116 Kb plasmid and five other strains (NCTC 8678, CP-35, NCTC 8449, CP-12 and 1001175st1_F9) in which the gene was absent. The clonal genomic relationship of chromosomal *cpe* strains found here was also recently described for *C.* *perfringens* strains from food poisoning cases in France^19^ and the United Kingdom^20^. Interestingly, all these studies reported the absence of *cpe* in a few strains within this phylogroup. Kiu et al^20^ pointed to the possibility of *cpe* gene loss as indicated by previous PCR results. A remarkable feature of this phylogroup was the high frequency of a limited number of insertion sequences as well as the relatively small genome size compared to other phylogroups (Fig. 3A, Supplementary Table S4). The high number of insertion sequences was especially observed in 18 genomes sequenced using Pacific Bioscience technology where the strains were represented by less than 30 contigs. 13 strains with highly fragmented genomes (79–252 contigs) showed a lower number of IS copies. The presence of many copies of IS elements may interfere with the proper assembly of genomes sequenced using short-read sequencing methods, and therefore the actual number of IS elements in these strains may be underestimated.

Expansion of IS elements together with a reduction in the genome size has been reported in different bacteria in association with bacterial specialization to certain niches for example in *Bordetella pertussis*, *Yersinia pestis* and *Shigella* species ^43–45^. Similarly, IS elements mediated genome decay but also gene duplication in the horse-restricted pathogen *Streptococcus equi* during the persistent infection phase ^46^. *C.* *perfringens* phylogroup I strains cause human foodborne illnesses ^4,47^. It has been proposed that chromosomal *cpe* strains have a different epidemiology and are adapted to an environment that differs from that of other *C.* *perfringens* strains ^5^. It is therefore plausible to assume that IS elements might drive the evolution of these strains towards a certain niche i.e., to replicate in the food matrix.

It has to be mentioned that some strains (JGS1721, JGS1495 and JXJA17) which are not members of phylogroup I also had high numbers of IS elements (Supplementary Table S3) indicating that the feature of IS expansion is not strictly limited to this phylogroup.

We also observed that most genomes of this phylogroup carry extrachromosomal elements and that none of these genomes except NCTC 8081 and CP-35 harbour the *tcp* conjugation locus (Fig. 3A, Supplementary Table S4).

Phylogroup I also included a Darmbrand strain which is genetically related to the chromosomal *cpe* strains as determined in this study based on whole-genome sequencing and in previous studies using classical MLST analysis ^5,47^. The historic Darmbrand strain involved in fatal outbreaks of necrotic enteritis in humans in Germany in the 1940s was unusually enriched with insertion sequences that account for 6% of the size of the chromosome (Fig. 3A, Supplementary Table S4). The insertion sequences also bordered and probably mediated several rearrangements of the chromosomal blocks in this strain (Fig. 1). Additionally, the Darmbrand strain carried six extrachromosomal elements, two of which contained the genes of the typing toxins (beta and enterotoxin, each on a separate plasmid) and the *tcp* conjugation locus (Table 1). In congruence with previous reports, MLST analyses based on eight housekeeping genes grouped phylogroup I strains with chromosomal *cpe* and Darmbrand strains together (Supplementary Table S5, Supplementary Fig. S4). In summary, these results corroborate the genetic relatedness between chromosomal *cpe* strains (and Darmbrand strains) suggesting a common evolutionary history as hypothesized before ^47^.

**Figure 5:**
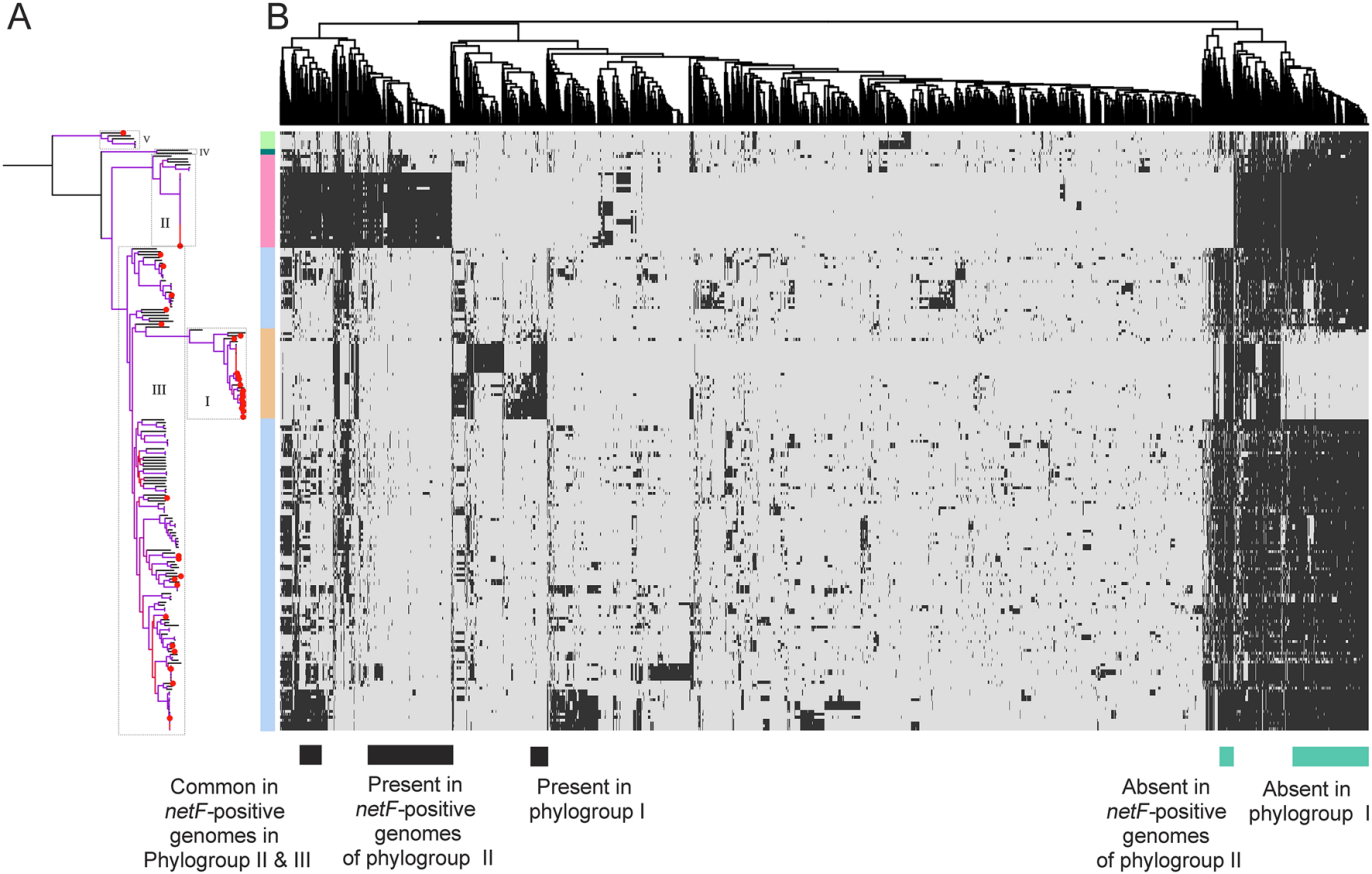
Association between core-genome SNPs and accessory gene content of 206 *Clostridium perfringens* genomes. (**A**) A maximum-likelihood phylogenetic tree based on core genome SNPs as presented in Fig. 3A with phylogroups being highlighted, shown as a coloured band next to the ML phylogeny. The phylogenetic tree was visualized using iTOL^81^ (**B**) A heat map showing the distribution of the 4,099 accessory genes (genes shared by 2—95% of the genomes). Rows represent the accessory genes present in each genome while columns represent accessory gene families. Black and light grey colourations specify accessory gene presence and absence, respectively. Accessory genes were clustered as indicated by the dendrogram (top) using clustering functions from the ComplexHeatmap R package. Genes specifically present or absent in different groups are highlighted (bottom). Heat maps were generated in R using the package ComplexHeatmap ^87,91^. The figure was edited in Adobe Photoshop CS5 (www.adobe.com/photoshop).

Phylogroup II contained in total 32 strains, including two strains of type A isolated from a human and a mouse and three strains of type E recovered from human and a cattle as well as a type F strain from a pig. The other 26 strains (Fig. 4A) were involved in cases of foal necrotizing enteritis and canine hemorrhagic diarrhoea. They carried a plasmid-encoded *netF* gene and a plasmid-encoded *cpe* gene ^25,48^. One strain of this group had a completed genome (strain JF838, Supplementary Table S2) which showed a larger size (3.5 Mb) and many plasmids (n = 5). Some of the plasmids carried the conjugative *tcp* locus (Supplementary Table S2) ^48^. Plasmids of this phylogroup were also detected in a cluster of six strains that belong to phylogroup III, also isolated from foal necrotizing enteritis and canine hemorrhagic diarrhoea. The finding that *netF*-genomes could split into two lineages was reported previously by Gohari and colleagues ^25^, hypothesizing that both lineages may have a common ancestor. However, we identified only 33 accessory genes that were consistently present in both *netF* lineages and absent in 90% of the other strains (Fig. 5, Supplementary Table S6). The genetic distance and the relatively small amount of common accessory genes between both lineages indicate a central role of plasmid-driven horizontal gene transfer for the virulence and clinical picture. The role of *C. perfringens* plasmids in virulence has recently been demonstrated for example in chicken isolates ^49^.

Phylogroup III is the largest and most heterogeneous group of *C. perfringens* that includes 135 strains from different hosts and involved in different diseases (Fig. 3). Strains of phylogroup III belong to all seven toxinotypes and carry the six typing toxin genes of *C.* *perfringens*. This is in contrast to phylogroup I in which the genes of the toxins NetB, ɛ and ɩ were not detected and phylogroup II in which the *netB* and ɛ toxin genes were not detected. Phylogroups IV and V were less abundant, including two and six strains, respectively.

Since the accessory gene profiles were to some extent in congruence with the core genome phylogeny (Figs. 3, 4), we aimed to identify accessory genes that are distinctly associated to different phylogroups and thus may contribute to the characteristic phenotype of some strains like disease outcome (Fig. 5). In phylogroup I, 90% of the strains lacked 233 chromosomal genes which were present in 90% of the phylogroup II and III strains (Fig. 5, Supplementary Table S7). In parallel, 90% of the strains of phylogroup I carried 35 additional gene families which were absent in 90% of the other strains. The pattern of gene loss (233 chromosomal genes) in phylogroup I was more prominent than gene gain (35 genes) which correlates well with the characteristic smaller genome size. The loss of chromosomal genes in phylogroup I was in sharp contrast to the *netF*-positive strains of phylogroup II where additional 292 genes and simultaneous absence of 21 chromosomal genes were found in 90% of these phylogroup II genomes but not in the other phylogroups (Supplementary Table S8). This indicates that the gain and loss of genetic elements within the species *C.* *perfringens* is not balanced in the different phylogroups. It seems that phylogroup II is directed to gain new genetic material while phylogroup I is directed toward gene loss. To the authors’ information, such a divergent pattern of evolution within a single species has not been described in other bacteria to date. A list of the identified genes and their distribution and function is provided in Supplementary Tables S7 and S8. In silico functional COG annotation of these genes revealed possible differences in the metabolic fitness between the phylogroups (Supplementary Table S9). 102 genes involved in metabolic functions such as carbohydrate, amino acid and energy production were missing in phylogroup I (Supplementary Table S9). In contrast, phylogroup II acquired 41 genes encoding for “cellular processes and signaling”, notably “cell wall/membrane/envelope biogenesis” (n = 16) as well as 41 genes involved in metabolism, possibly enhancing the fitness of the strains for host colonization.

In summary, it can be hypothesized that the pronounced distribution pattern of accessory genes, which is also reflected in the phylogeny of the core genome, could possibly be correlated with the adaptation of the strains to certain host niches, especially in the case of phylogroup I.

### *C. perfringens* has a large repertoire of potential virulence factors

**Figure 6:**
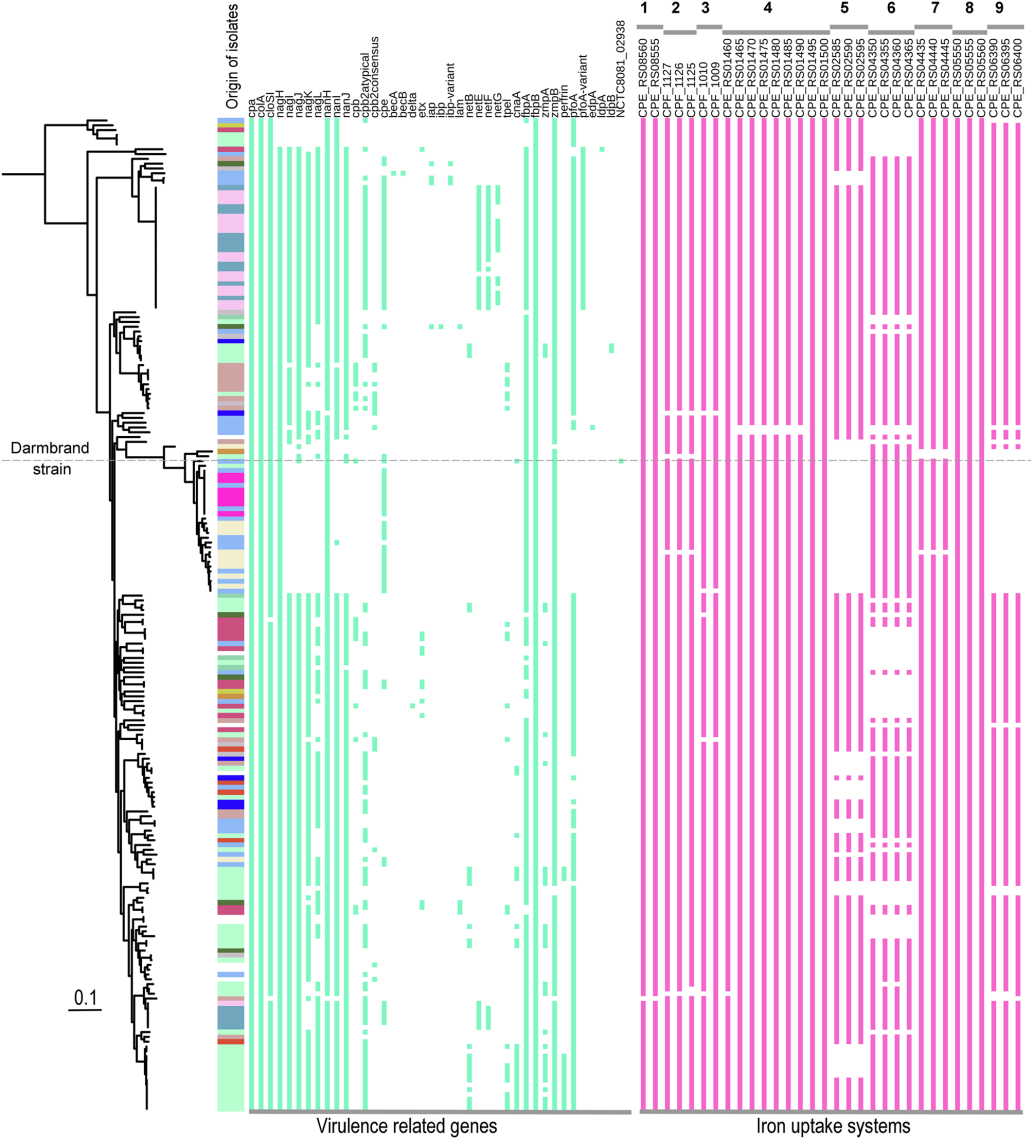
Distribution of virulence-related genes and putative iron uptake systems in the 206 *Clostridium perfringens* genomes. The presence and absence of genes are presented next to the maximum likelihood phylogeny with the origin of isolates being depicted, similar to Fig. 3A. Coloured cells denote the gene presence and white denotes gene absence. The dotted horizontal line refers to the virulence profile of the Darmbrand strain. The locus tag NCTC8081_02938 describes the toxin gene homolog found in the Darmbrand strain. The iron uptake systems are numbered as follow: *feoAB* operon for ferrous iron-acquisition systems (numbered 1 to 3), two putative heme acquisition systems (4 and 5), one putative ferric citrate iron acquisition system (6) and three putative siderophore iron acquisition systems (7 to 9). The column headers for the iron uptake systems are locus tags of genes from strain 13 [1 and 4 to 9] and ATCC 13124 [2 and 3]. The figure was produced using iTOL ^81^.

Using the 206 genomes, we searched for previously described virulence-related genes in *C. perfringens* (n = 77 genes, see methods, Supplementary Table S10 and Data set 5 at https://doi.org/10.6084/m9.figshare.12264497). The results showed a distinct pattern of distribution of the virulence genes between phylogroups possibly reflecting different pathogenic potential of strains (Fig. 6, Supplementary Table S11). The chromosomally-encoded alpha-toxin (*cpa* or *plc*) and collagenase (*colA*) genes were present in all strains, followed by the gene for alpha-clostripain (*closI* or *ccp*) found in 99% of the strains. The *colA* gene was however truncated in two genomes that had a large deletion mutation (Data set 6 at https://doi.org/10.6084/m9.figshare.12264497). The sialidase genes *nanH*, *nanI* and *nanJ* were present at a frequency of 99.5%, 84.4% and 82%, respectively, with *nanI* and *nanJ* being absent in most strains of phylogroup I (28 out of 31) and *nanJ* being absent in all strains of phylogroup V (Fig. 6, Supplementary Table S11). Similarly, the μ-toxin (hyaluronidase) genes (*nagHIJKL*) were absent in phylogroup V while most phylogroup I strains (28 out of 31) lacked the μ-toxin genes (*nagIJKL*) and harboured a truncated *nagH* gene with a large deletion mutation (Supplementary Table S11 and Data set 7 at https://doi.org/10.6084/m9.figshare.12264497). Sialidases enhance bacterial colonization of the intestinal tract and promotes the cytotoxicity of *C.* *perfringens* while μ-toxin degrades hyaluronic acid in the connective tissue and facilitates the spread of *C. perfringens* toxins ^50–52^. The absence of these genes in phylogroup I is in agreement with previous findings of limited production of sialidases by chromosomal *cpe* strains ^53^. The authors suggested that NanI can be dispensable during the usual acute course of diseases induced by these strains ^53^. It is worth mentioning that only one strain in phylogroup I carried the *nanI* gene, and that the Darmbrand strain and strain NCTC 10240 had each a *nanJ* exo-sialidase gene as well as an intact *nagJ* μ-toxin gene (Fig. 6, Supplementary Table S11).

The gene encoding perfringolysin O (*pfoA*)^54^ was absent in all strains of phylogroup I and IV. 15 strains from phylogroup III did also not carry the *pfoA* gene. Interestingly, a variant for the *pfoA* gene with 85.7% nucleotide identity and 81.7% amino acid (aa) identity to the typical *pfoA* gene was found exclusively in phylogroup IV and II (Fig. 6, Supplementary Table S11). This variant was located downstream of the *pfoA* gene in strain JP838 (Data set 8 at https://doi.org/10.6084/m9.figshare.12264497).

Recently, Lacey et al. 2019 ^55^ described eight novel toxin gene homologs that were associated with mobile elements in *C. perfringens*. Three of these genes were found in the data set under study; the *edpA* gene encoding a homolog with an epsilon toxin-like aerolysin domain (n = 1 strain), and *ldpA* (n = 1 strain) and *ldpB* (n = 2 strain) with a leucocidin/hemolysin domain (Fig. 6, Supplementary Table S11). We further identified a hitherto unknown toxin homolog with a leucocidin/hemolysin domain that was present only in the Darmbrand strain (Fig. 6, Supplementary Table S11) and had 65.5% and 50.5% aa identity to NetG and β toxin genes, respectively. The toxin homolog, flanked by the insertion sequences IS*1469*, IS*Cpe2* and IS*1470*, was located on a large plasmid (size = 116 Kb) which also carried the *cpe* gene and *tcp* locus, suggesting that this plasmid could be a conjugative plasmid ^56^. This plasmid was distinct from a *cpb* carrying plasmid (size = 67.9 Kb) additionally present in the Darmbrand strain (Data set 9 at https://doi.org/10.6084/m9.figshare.12264497). The role of the identified toxin homolog for the virulence of the Darmbrand strain remains to be elucidated. Moreover, unlike chromosomal *cpe* strains*,* the virulence profile of the Darmbrand strain included a *cnaA* gene, a gene recently found to enhance the adherence of necrotic enteritis strains to collagen and was linked to the increased pathogenicity of *C. perfringens* in poultry ^57,58^ (Fig. 6).

Prior analysis of the genome of *C.* *perfringens* strain 13 identified seven putative iron-acquisition systems: two heme-acquisition systems, one ferrous iron-acquisition system (*feoAB*), three siderophore-mediated acquisition systems and one ferric citrate iron-acquisition system ^59^. Strains ATCC 13124 and SM101 were also reported to have three and two copies of *feoAB* operon, respectively ^41^. Two of these systems (ferrous iron-acquisition system encoded by *feoAB* operon and a heme-uptake system encoded by *C.* *perfringens* heme transport “*Cht*” locus) were experimentally proven to be essential for the virulence of *C.* *perfringens* in gas gangrene models ^59,60^. Both loci (*Cht* and *feoAB*) were present in almost all investigated *C.* *perfringens* genomes (99% presence) (Fig. 6, Supplementary Table S11). The additional two copies of *feoAB* identified in the type strain ATCC 13124 were detected at a frequency of 98% (Fig. 6, Supplementary Table S11). Further putative iron acquisition systems were observed in more than 70% of the strains (Fig. 6, Supplementary Table S11). The three siderophore-based systems in strain 13 were found respectively at a frequency of 98%, 100% and 80% while the ferric citrate iron acquisition system was present in 76% of the strains (Fig. 6, Supplementary Table S11). Interestingly, one heme- and one siderophore-iron uptake system were absent in phylogroup I strains while the ferric citrate iron acquisition system was absent in phylogroup V. In addition, there was mostly a general 100% gene linkage within each of these systems i.e., all genes were present or absent. Taken together, the preservation of a variety of iron uptake systems in *C.* *perfringens* could enable the bacterium to survive iron shortage conditions in various situations and to retrieve iron sequestered by host proteins during infections. However, it was intriguing that two of these iron systems were missing throughout all the phylogroup I strains.

To further explore potential virulence landscape in *C. perfringens*, we clustered the protein sequences of the 206 genomes at 90% BLASTP identity and searched these protein clusters against the core protein set of virulence factor database using BLASTP (see methods). The in silico prediction identified a vast arsenal of additional 510 genes potentially linked to adaptation and pathogenicity in *C. perfringens* (Supplementary Table S12, (Data set 10 at https://doi.org/10.6084/m9.figshare.12264497). The presence of these genes was different between the phylogroups, with phylogroup I carrying less virulence gene homologs (average 141 ± 5 genes) compared to other phylogroups II (average 166 ± 3 genes), III (average 160 ± 7 genes), IV (average 166 ± 1 genes) and V (average 149 ± 4 genes) (Supplementary Table S12). 157 genes (30%) of the identified 510 gene homologs showed 23–84% aa identity to known capsular genes in Gram-positive and Gram-negative bacteria (Supplementary Table S13). The number of the capsular gene homologs varied between 12 and 26 for all strains with minor variations between phylogroups. 9% of these homologs were present in all 206 strains (core capsular genes) while 90% were part of the variable genome (Supplementary Table S13), indicating a variable capsular structure. However, we cannot exclude the possibility that strongly divergent sequences for capsule genes remained undiscovered. Previous studies reported the presence of capsular genes in genomic islands ^41^. A recent in silico study described highly diverse capsule types in *C.* *perfringens* from poultry as a probable virulence factor with roles in colonization and immune evasion ^22^.

## Conclusion

This study provides new insights into the genomic variability and phylogenetic structure of *C.* *perfringens*, a typical inhabitant of the environment and digestive tract of many species including humans. Utilizing 206 public available genomes of *C.* *perfringens* strains from diverse ecological niches, we gained insight into the phylogeny of this globally important pathogen. Our analysis unravelled five stable phylogroups. This has been very recently confirmed in parallel by other workers ^61^ after this article was submitted for review. Phylogroup I strains are mainly involved in human foodborne illness and exhibit unique genomic characteristics such as the high presence of insertion sequences and excessive loss of genes involved in metabolism and virulence. Similar features were reported in other bacteria where evolution has led to bacterial specialization toward a certain habitat such as *Steptococcus equi* ^46^ and *Shigella* species ^45^. The loss of genes in this phylogroup contrasts most strains (26 out of 32) of phylogroup II that were isolated from enteric lesions in horses and dogs which appear to be directed towards gaining new genetic material. In summary, our data showed that even in a spore-forming species like *C. perfringens*, the occupation of certain habitats could have a strong influence on phylogeny. The data presented here provide new genomic framework and impetus for future studies to investigate ecological niche adaptation and diversification of this important pathogen.

## Materials and methods

### Data acquisition and assembly

The publicly-available sequence data of *C. perfringens* totalling 206 genomes were included in the study. These data comprised 23 raw Pacific-Bioscience data available from the NCTC 3000 project ^23^ as well as 183 (out of 205) genome assemblies available at the NCBI (Data set 1 and 2 at https://doi.org/10.6084/m9.figshare.12264497). For the NCBI genomes, we estimated the average nucleotide identity using pyani v0.2.3 software ^62^ and excluded genomes with less than 95% concordance as well as 18 duplicated genomes. The Pacific-Bioscience sequence data of 23 *C.* *perfringens* NCTC strains were de novo assembled using RS_HGAP_Assembly v3 via SMRT Analysis system v2.3.014 ^63^. For the strain NCTC 8081, canu v1.6 ^64^ was used instead of HGAP, as we observed a continuous merge of the plasmid to the chromosome. The corrected preassembled reads from HGAP were exported and used as input for canu v1.6 (parameter: correctedErrorRate = 0.075). The circularization protocol was performed as follow: first, we used Gepard ^65^ to identify similar parts at the ends of each contig. Identified overlapping ends were merged and the genomes were circularized using Circlator ^66^ or check_circularity.pl from SPRAI (available from Hunt et al. 2015 ^66^). Errors in the merged region were iteratively refined with Quiver algorithm (RS.Resequencing.1) resulting in contigs with at least 99.99% concordance to the reference (Data set 1 at https://doi.org/10.6084/m9.figshare.12264497).

### Genome annotation and comparison

Genome annotation was performed using Prokka v1.13.3 ^67^ and Rapid Annotation using the Subsystem Technology (RAST) ^68^. Insertion sequences were predicted using ISEscan v1.7.2 ^69^. Prophage and genomic islands prediction were performed using PHASTER ^70^ and Islandviewer4 ^71^, respectively. CRISPR prediction was performed using the CRISPR Recognition Tool v1.1 ^72^. Genome comparison was carried out using progressiveMauve ^73^.

The in silico MLST was performed according to Deguchi et al., 2009 ^74^. Briefly, MLST genes were searched in the WGS data using BLASTN v2.9.0 + ^75^. 187 genomes in which MLST genes were detected were then processed using custom scripts to extract MLST sequences. Additionally, classical MLST data from another 71 strains (Supplementary Table S5) investigated and published in prior studies were included ^17,47,74^. MAFFT v7.307 ^76^ was used for alignment (option–auto) and a neighbor joining tree was constructed using MEGA X ^77^with 500 bootstrap support and gap sites being removed (option complete deletion). The final MLST tree (Supplementary Fig. S4) was based on 5.1 Kb sequence alignments.

### Core genome phylogeny

A core genome alignment was performed using Parsnp v1.2 with default parameters ^78^. RAxML v8.2.10 was then used to construct a maximum-likelihood phylogenetic tree with general time-reversible (GTR)-gamma model and 100 bootstrap replicates ^79^. Clades were assigned using RAMI based on patristic distance (sum of branch length, with threshold = 0.01) ^80^. The phylogenetic tree was visualized using iTOL ^81^. SplitsTree4 ^82^ was used with the core genome SNPs to infer phylogenetic network using a NeighbourNet method with the Uncorrected P model of substitution ^83^. The average nucleotide identity was performed using pyani v0.2.3 ^62^.

### Gene content analysis

A pangenome was constructed using Roary v3.12.0 ^84^ at 90% BLAST identity (-i 90) and enabled paralogues clustering (Data set 4 at https://doi.org/10.6084/m9.figshare.12264497). Genes found in 2–95% of the genomes were defined as accessory genes. The species accumulation curve as well as Jaccard distances between accessory gene profiles were calculated as described ^85^, using *vegan* ^86^ in R ^87^. A multidimensional scaling plot of the pangenome was calculated in R using the cmdscale () function.

### In silico identification of virulence-related genes

BLAST analysis was performed to search the 206 genomes for the presence of virulence and pathogenicity related genes. First, we created a custom database including the up-to-date virulence factors described in the literature on *C. perfringens* (Supplementary Table S9). Then, we searched for the presence of these virulence factors in the 206 strains using BLASTN via ABRicate v1.0.1 (https://github.com/tseemann/abricate) with 90% identity and 30% coverage.

Next, we searched for potential homologous genes that might be related to the virulence of *C. perfringens*. For that, we performed BLAST analysis of the clustered protein sequences from the 206 strains against the core protein set of the virulence factor database (VFDB set A) ^88^. This database represents the experimentally verified virulence factors from various pathogens. As thresholds we used e-value < 1e−20 and query alignment length > 70% ^89^. BLAST hits more than 20% identity at the protein level were reported. Annotation of the identified data set was performed using OmicBox (www.biobam.com/omicsbox) and the COG ^90^ database.

## Supplementary Information

Supplementary Information 1.

Supplementary Information 2.

## Data Availability

The datasets generated during and/or analysed during the current study are available in the figshare repository, https://doi.org/10.6084/m9.figshare.12264497.
