# Supplementary material for: Comparative in silico genome analysis of Clostridium perfringens unravels stable phylogroups with different genome characteristics and pathogenic potential
Source: Sci Rep. 2021 Mar 24;11:6756. doi: 10.1038/s41598-021-86148-8 (PMC7991664; doi:10.1038/s41598-021-86148-8)
Supplement: Supplementary file 1 — Supplementary Information 1. [file 41598_2021_86148_MOESM1_ESM.pdf]

Supplementary Material Figures S1 – S4

Comparative *in silico* genome analysis of *Clostridium perfringens* unravels stable phylogroups with different genome characteristics and pathogenic potential

Mostafa Y. Abdel-Glil[1,2*], Prasad Thomas[1#], Jörg Linde[1], Anne Busch[1§], Lothar H. Wieler[3,4], Heinrich Neubauer[1], Christian Seyboldt[1*]

[1]Institute of Bacterial Infections and Zoonoses, Friedrich-Loeffler-Institut, Naumburger Str. 96A, 07743 Jena, Germany.

[2]Department of Pathology, Faculty of Veterinary Medicine, Zagazig University, Sharkia province, Egypt.

[3]Robert Koch-Institut, Nordufer 20, 13353, Berlin, Germany.

[4]Institute of Microbiology and Epizootics, Department of Veterinary Medicine, Freie Universität, Robert-von-Ostertag-Str. 7-13, Building 35, 14163, Berlin, Germany.

*Address correspondence to M. Y. Abdel-Glil: mostafa.abdelglil@fli.de and C. Seyboldt: christian.seyboldt@fli.de

Content

Figure S1: A genome alignment of the chromosome of 34 *Clostridium perfringens* strains using Mauve.

Figure S2: The distribution of mobile genetic elements in the closed chromosome of 34 *Clostridium perfringens* genomes.

Figure S3: A maximum likelihood (ML) phylogeny computed from the core genome SNPs.

Figure S4: MLST phylogenetic tree of 258 *C. perfringens* strains based on eight MLST housekeeping genes.

Figure S1: A genome alignment of the chromosome of 34 *Clostridium perfringens* strains using Mauve. Chromosomal regions were arranged relative to the type strain ATCC 13124. Homologous blocks (Locally Collinear Blocks; LCBs) are of the same color and linked between the strains. Coordinate 1 in each genome corresponds to the origin of replication. A total of 56 LCBs of size ranging between 243bp to 389.5Kb were present in all 34 strains.

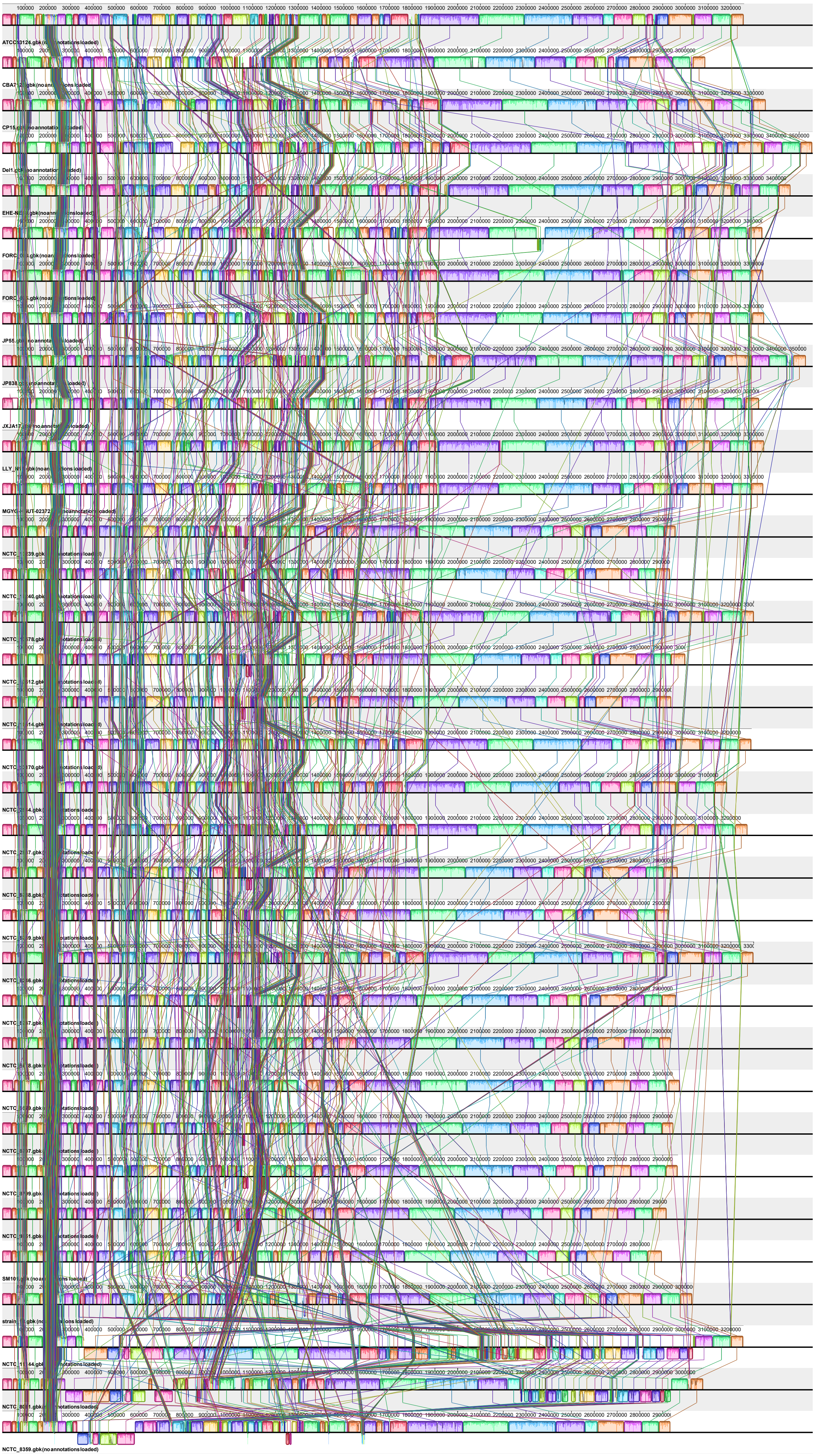
NCTC_8359.gbk(no annotations loaded)

Figure S2: The distribution of mobile genetic elements in the closed chromosome of 34 *Clostridium perfringens* genomes. The distribution of the insertions sequences (IS) (first line) and genomic islands (GI) (second line) across the chromosome in the closed genomes of *C. perfringens*. An uneven distribution of IS and GI in the chromosomal *cpe* strains with an accumulation in the first chromosomal half was seen (left). The genome of the Darmbrand strain NCTC 8081 (middle) was also rich in the IS- and GI, an uneven distribution of IS and GI toward a chromosomal half was not observed. Note the different scaling of y-axes. Position 1 represents the origin of replication as previously determined in strain 13.

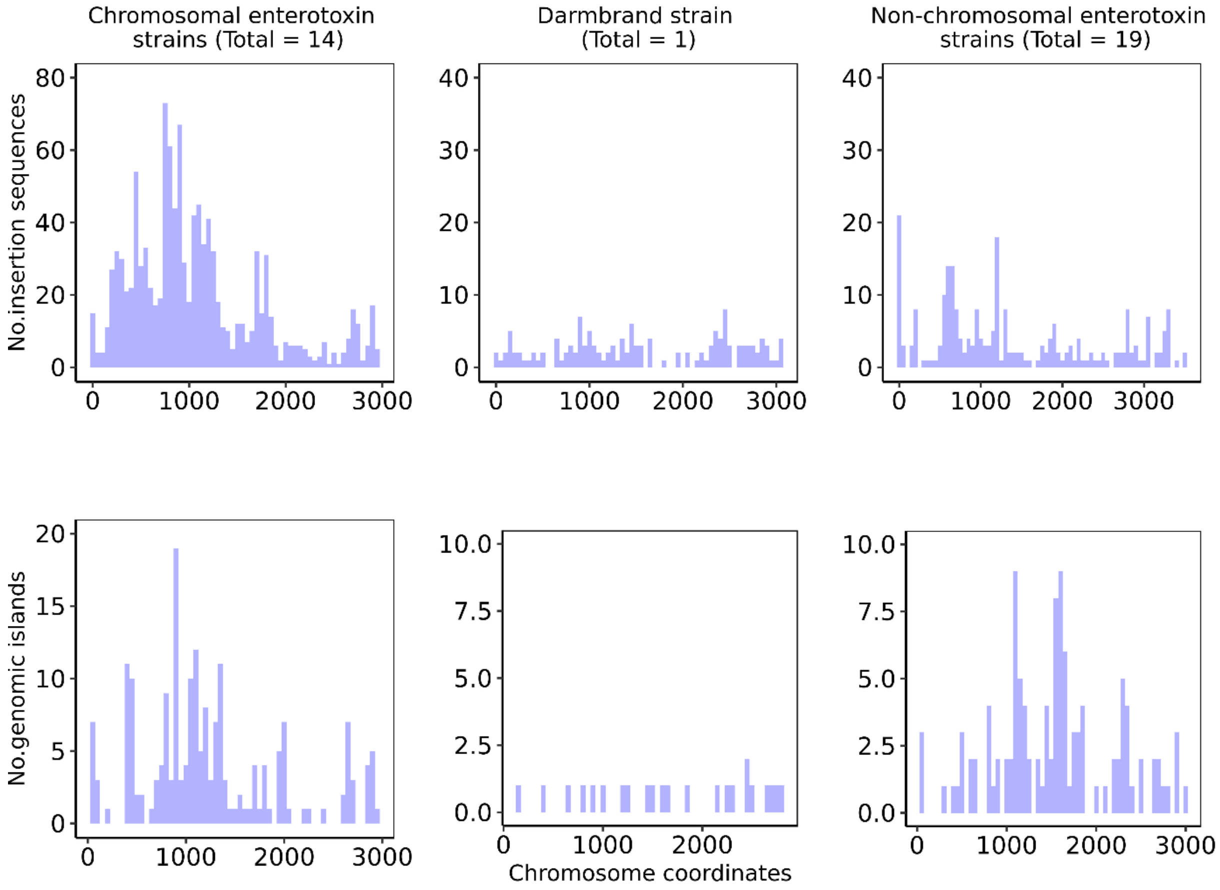
Chromosomal enterotoxin strains (Total = 14)
Darmbrand strain (Total = 1)
Non-chromosomal enterotoxin strains (Total = 19)
No.insertion sequences
No.genomic islands
Chromosome coordinates

Figure S3: A maximum likelihood (ML) phylogeny computed from the core genome SNPs as presented in figure 3A with clusters (n=118 clusters) identified based on patristic distance using RAMI tool being highlighted.

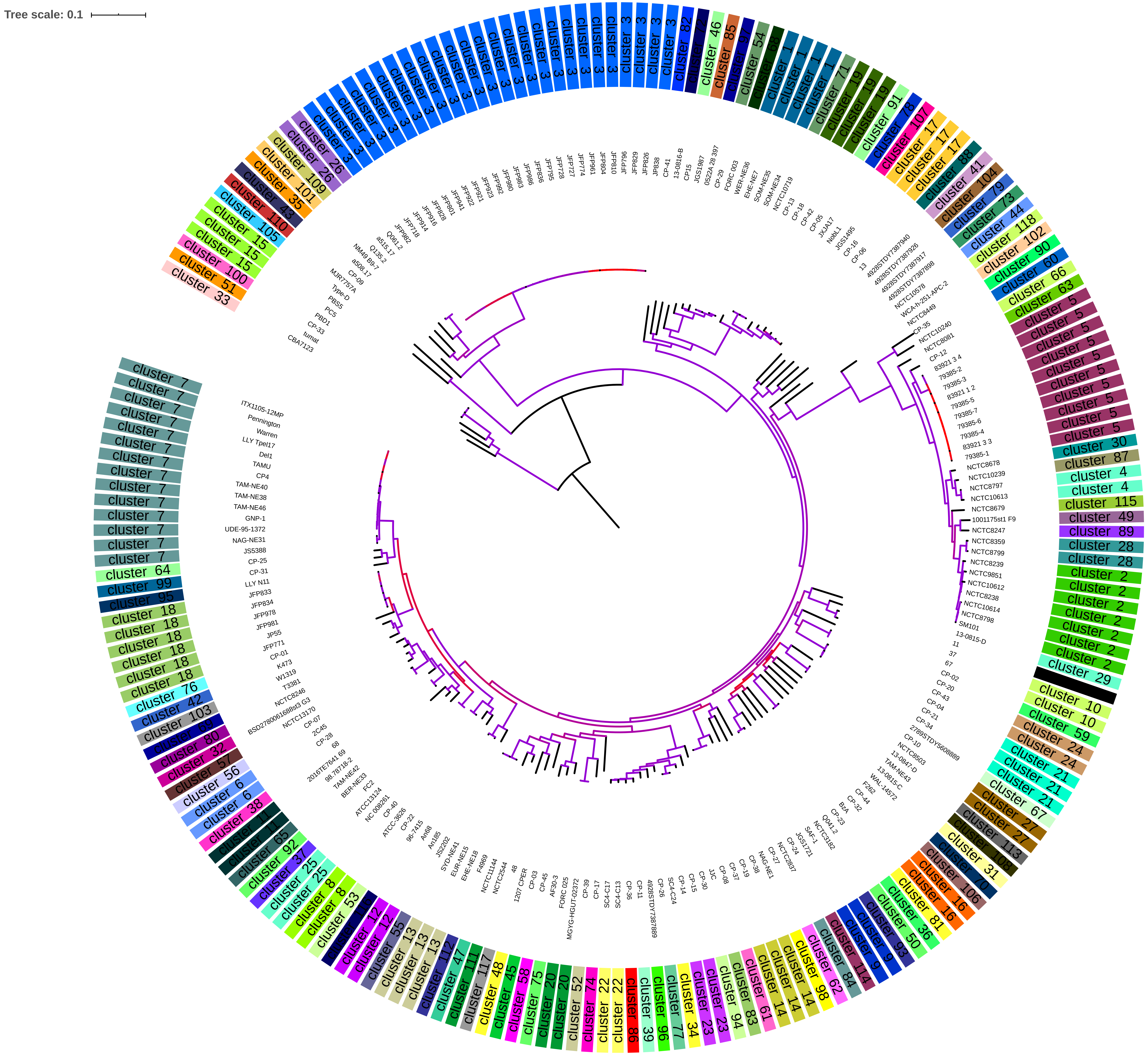
Tree scale: 0.1

Figure S4: MLST phylogenetic tree of 258 *C. perfringens* strains based on eight MLST housekeeping genes. MLST loci were found in whole genome sequence data of 187 strains investigated in this study. These loci were *in silico* extracted and compared to MLST data from strains investigated in prior studies (Deguchi et al., 2009, Ma et al., 2012 and Xiao et al., 2012). Note that the food poisoning strains that carry chromosomal *cpe* and the human enteritis necroticans strains were grouped together (52 strains) and formed a distinct phylogenetic branch as highlighted. Branch coloration denotes bootstrap values as in Figure 3.

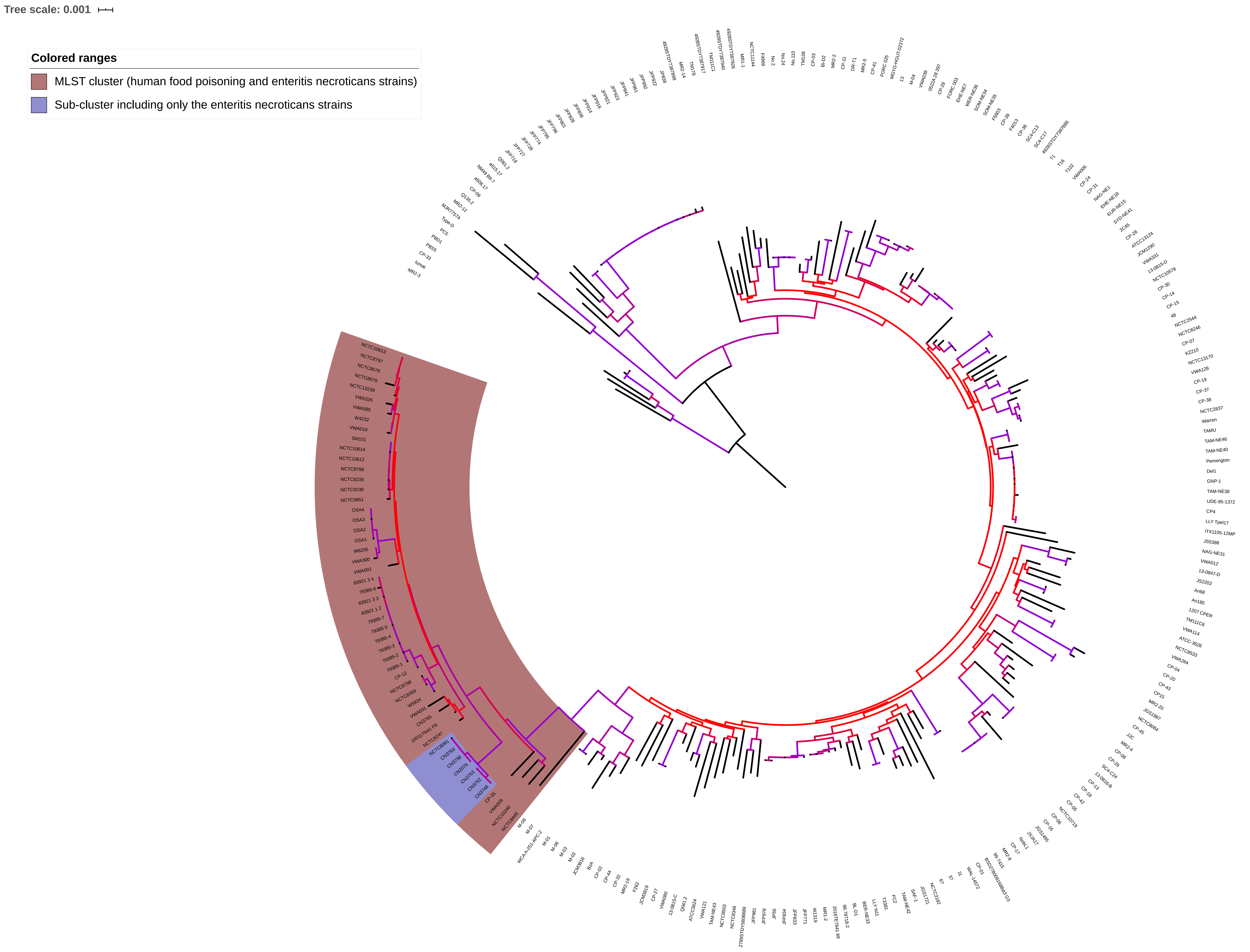
Tree scale: 0.001
Colored ranges
MLST cluster (human food poisoning and enteritis necroticans strains)
Sub-cluster including only the enteritis necroticans strains
